# Cross-talk of MLST and transcriptome unveiling antibiotic resistance mechanism of carbapenem resistance *Acinetobacter baumannii* clinical strains isolated in Guiyang, China

**DOI:** 10.3389/fmicb.2024.1394775

**Published:** 2024-06-14

**Authors:** Zhilang Qiu, Kexin Yuan, Huijun Cao, Sufang Chen, Feifei Chen, Fei Mo, Guo Guo, Jian Peng

**Affiliations:** ^1^Center for Clinical Laboratories, The Affiliated Hospital of Guizhou Medical University, Guiyang, China; ^2^Key Laboratory of Infectious Immune and Antibody Engineering of Guizhou Province, Cellular Immunotherapy Engineering Research Center of Guizhou Province, School of Biology and Engineering/School of Basic Medical Sciences, Guizhou Medical University, Guiyang, China; ^3^The Key and Characteristic Laboratory of Modern Pathogen Biology, Basic Medical College, Guizhou Medical University, Guiyang, China; ^4^Translational Medicine Research Center, Guizhou Medical University, Guiyang, China

**Keywords:** carbapenem resistance *A. baumannii*, MLST typing, antibiotic resistance, antibiotic resistance mechanism, transcriptome

## Abstract

**Introduction:**

*Acinetobacter baumannii* (*A. baumannii*) is an important opportunistic pathogen causing nosocomial infection in the clinic. The occurrence rate of antibiotic resistance is increasing year by year, resulting in a highly serious situation of bacterial resistance.

**Methods:**

To better understand the local epidemiology of multidrug-resistant *A. baumannii*, an investigation was conducted on the antibiotic resistance of different types of *A. baumannii* and its relationship with the genes of *A. baumannii*. Furthermore, the molecular mechanism underlying antibiotic resistance in *A. baumannii* was investigated through transcriptome analysis.

**Results:**

These results showed that a total of 9 STs were detected. It was found that 99% of the strains isolated in the hospital belonged to the same STs, and the clone complex CC208 was widely distributed in various departments and all kinds of samples. Furthermore, these *A. baumannii* strains showed high resistance to ertapenem, biapenem, meropenem, and imipenem, among which the resistance to ertapenem was the strongest. The detection rate of *bla*_*OXA*–51_ gene in these carbapenem resistance *A. baumannii* (CRAB) reached 100%; Additionally, the transcriptome results showed that the resistance genes were up-regulated in resistance strains, and these genes involved in biofilm formation, efflux pumps, peptidoglycan biosynthesis, and chaperonin synthesis.

**Discussion:**

These results suggest that the CC208 STs were the main clonal complex, and showed high carbapenem antibiotic resistance. All these resistant strains were distributed in various departments, but most of them were distributed in intensive care units (ICU). The *bla*_*OXA*–23_ was the main antibiotic resistance genotype; In summary, the epidemic trend of clinical *A. baumannii* in Guiyang, China was analyzed from the molecular level, and the resistance mechanism of *A. baumannii* to carbapenem antibiotics was analyzed with transcriptome, which provided a theoretical basis for better control of *A. baumannii*.

## 1 Introduction

The Gram-negative bacterium *Acinetobacter baumannii* (*A. baumannii*) is known as one of the most important nosocomial pathogens in healthcare-associated infections, especially with ventilator-associated pneumonia and catheter-associated infections (Liu et al., 2022; Kang et al., 2023). Additionally, *A. baumannii* has been reported to be associated with high mortality in intensive care units (ICUs), particularly among critically ill patients (Inchai et al., 2015). In 2017, The World Health Organization (WHO) also recently listed ESKAPE pathogens in the list of 12 bacteria against which new antibiotics are urgently needed (Mulani et al., 2019; Wang et al., 2022). Overuse of antibiotics has prompted the emergence of carbapenem resistance *A. baumannii* (CRAB), in consequence, either horizontal gene transfer or other opportunities, such as altered targets, decreased membrane permeability (Chusri et al., 2014), increased production of degrading enzymes (Lee et al., 2017), overexpression of efflux pumps (Roy et al., 2021), metabolic changes, biofilm formation (Yan and Bassler, 2019; Colquhoun and Rather, 2020; Gedefie et al., 2021), or increased nutrient sequestration mechanisms (Tiwari et al., 2019).

Antimicrobial resistance is a prominent feature that helps *A. baumannii* survive in an antimicrobial stress environment, causing the microorganism to pose a threat to humans and complicating treatments (Han et al., 2022). Multidrug-resistant *A. baumannii*, extensively drug-resistant *A. baumannii*, and pan-drug-resistant *A. baumannii* nosocomial isolates are now commonly found in hospitals (Ramirez et al., 2020). Additionally, *A. baumannii* is also an emerging pathogen among the elderly and immunocompromised patients in nursing homes and community hospitals (Mody et al., 2015).

The rise of resistant *A. baumannii* strains prompted the design and execution of epidemiological investigations of *A. baumannii* epidemics using various molecular typing methods, including multi-locus enzyme electrophoresis (MLEE), pulsed-field gel electrophoresis (PFGE), and subsequently confirmed by multi-locus sequence typing (MLST) (Montanaro et al., 2016). Among these methods, the MLST has become the gold standard for epidemiological (Ravaioli et al., 2022). Furthermore, a prominent advantage of MLST is the derived nomenclature, and sequence type (ST), which has been rapidly and extensively adopted by the community, thus widening the global collective knowledge of the distribution, dissemination, and biological characteristics of crucial clonal populations (Gaiarsa et al., 2019).

A large number of studies have shown that genes play a vital role in the antibiotic resistance of any bacteria (Roy et al., 2022). It reported that a variety of OXA-type β-lactamase genes were found in CRAB strains (Abouelfetouh et al., 2022). Among these genes, the OXA-23 and OXA-51 were key determinants of carbapenem resistance (Chan et al., 2022). In addition, genes associated with efflux pumps also play an important role in carbapenem antibiotic resistance. It is reported that the Δ*aceI* mutant strain showed lower resistance to chlorhexidine than the parental strain. This result suggests that *aceI* plays a key role in antibiotic resistance (Liu et al., 2018). Besides, the gene *purF*, which regulates the biofilm formation, also plays a fatal role in antibiotic resistance (Dai et al., 2021). In addition, the Chaperonin GroEL/GroES also plays an important role in *Escherichia coli* antibiotic resistance (Goltermann et al., 2015). However, the mechanism of antibiotic resistance of *A. baumannii* needs to be further explored.

In the present study, the MLST, antibiotic resistance genes and transcriptome of *A. baumannii* were analyzed. This study found that a total of 9 ST sequence types were detected, and the CC208 was the main clonal complex in this hospital and showed high antibiotic resistance to carbapenems; The *bla*_*OXA*–23_ gene was the main antibiotic resistance genotypes; The transcriptome result suggests that carbapenem antibiotic resistance of *A. baumannii* involved biofilm formation, efflux pumps, peptidoglycan biosynthesis, and chaperonin synthesis.

## 2 Materials and methods

### 2.1 Bacterial strains

The 199 strains CRAB were isolated from patients in various departments of Affiliated Hospital of Guizhou Medical University, Guizhou, China from 2017 to 2019. Standard *A. baumannii* ATCC19606, pan-resistant *A. baumannii* strains are preserved in the Key Laboratory of Modern Pathogenic Biology, School of Basic Medicine, Guizhou Medical University.

The genomic DNA of *A. baumannii* was extracted using the kit (BIOTEKE, Beijing, China). Seven housekeeping genes were amplified by PCR. The primers used are listed in Table 1. The PCR reactions were performed using PrimeSTAR^®^ Max DNA Polymerase (Takara, Japan) with the following amplification parameters: 95°C for 5 min; 94°C for 30 s, 50°C for 30 s, and 72°C for 40 s, 30 cycles; 72°C for 10 min.

**TABLE 1:** Primers for PCR amplification of *A. baumannii* housekeeping genes.

Locus	Primer	Sequences	Amplicon size (bp)	Usage
*gltA*	CitratoF1	AATTTACAGTGGCACATTAGGTCCC	722	amp/seq
	CitratoR12	GCAGAGATACCAGCAGAGATACACG		amp/seq
*gyrB*	APRU F	TGTAAAACGACGGCCAGTGCNGGRTCYTTYTCYTGRCA	909	amp
	M13[-21]	TGTAAAACGACGGCCAGT		seq
	UP1E-R	CAGGAAACAGCTATGACCAYGSNGGNGGNAARTTYRA		amp
	M13-F	CAGGAAACAGCTATGACC		seq
*gdhB*	GDHB1F	GCTACTTTTATGCAACAGAGCC	775	amp
	GDHSECF	ACCACATGCTTTGTTATG		seq
	GDHB775R	GTTGAGTTGGCGTATGTTGTGC		amp
	GDHSECR	GTTGGCGTATGTTGTGC		seq
*recA*	RA1	CCTGAATCTTCYGGTAAAAC	425	amp/seq
	RA2	GTTTCTGGGCTGCCAAACATTAC		amp/seq
*cpn60*	CPN-3F2	ACTGTACTTGCTCAAGC	479	amp/seq
	CPN-R2	TTCAGCGATGATAAGAAGTGG		amp/seq
*gpi*	gpi_F	AATACCGTGGTGCTACGGG	508	amp/seq
	gpi_R	AACTTGATTTTCAGGAGC		amp/seq
*rpoD*	70F-RPOD	ACGACTGACCCGGTACGCATGTAYATGMGNGARATCGCNACNCT	492	amp
	70FS	ACGACTGACCCGGTACGCATGTA		seq
	70R RPOD	ATAGAAATAACCAGACGTAAGTTNGCYTCNACCATYTGYTTYTT		amp
	70RS	ATAGAAATAACCAGACGTAAGTT		seq

### 2.2 Multilocus sequence typing (MLST)

The amplified products of MLST typing of *A. baumannii* were sequenced, and the sequence results were compared on the MLST comparison website of *A. baumannii*. The sequencing results were submitted to the website for comparison to obtain allele number and ST type. The Bio Numerics 7.1 software is used for typing data processing, and generating minimum spanning tree (MST).

### 2.3 Detection of antibiotic susceptibility and resistance genes

The minimal inhibitory concentration (MIC) values of *A. baumannii* isolated from clinical isolates were measured by the micro-dilution broth method. The antibiotics determined were imipenem, meropenem, ertapenem, and biapenem. There were three repeats for each strain and antibiotic. Except for 5 gradients of biapenem, 6 gradients were done. The concentration of the mother liquid before dilution is shown in Table 2. The results were judged by the Institute of Clinical and Laboratory Standards (CLSI2012).

**TABLE 2:** The preparation of antibiotics.

Antibiotic	Solvent/diluent	Concentration of mother liquor before dilution (μg/mL)
Imipenem	1 × Phosphate buffer pH 7.2	2,048
Ertapenem	1 × Phosphate buffer pH 7.2	4,096
Meropenem	ddH_2_O	2,048
Biapenem	ddH_2_O	512

In this study, the *bla*_*OA*–23_ and *bla*_*OXA*–51_ were selected as molecular markers for rapid identification of *A. baumannii*. The primers are listed in Table 3, and the PCR reaction system is 15 μL, including the premixTaq enzyme 7.5 μL, the primers 1 μL with 10 μM, DNA template 1 μL, and the ddH2O 4.5 μL. Meanwhile, the PCRs were performed at 94°C for 5 min, followed by 35 cycles of 30 s at 94°C, 30 s at 55°C, 30 s at 72°C, and a final extension of 10 min at 72°C. The 5 μL PCR products were sampled in 1% agarose gel and 30 min was electrophoretic at a constant voltage of 100V. The photos were taken by WD-9413B gel imaging analyzer.

**TABLE 3:** Primers information for amplifying carbapenemase genes in *A. baumannii* strains.

Target gene	Primer	Primer sequence (5′ → 3′)	Product Size/bp	Temperature/°C
*blaOXA-23*	OXA-23-F	GATCGGATTGGAGAACCAGA	501	55
	OXA-23-R	ATTTCTGACCGCATTTCCAT		
*blaOXA-51*	OXA-51-F	ATGAACATTAAAGCACTCTTACTT	825	55
	OXA-51-R	CTATAAAATACCTAATTGTTCTAA		

### 2.4 RNA isolation, library construction and sequencing

The AB77 (weak imipenem resistance) and AB98 (strong imipenem resistance) were selected for transcriptome sequencing with three biological repeats per sample. After resuscitation of CRAB77 and CRAB98, respectively, 1 mL solution was absorbed and cultured in 100 mL LB liquid medium. After overnight incubation at 37°C 220 rpm until logarithmic growth, the organisms were collected by centrifugation, and three biological replicates were performed in each group, totaling six samples. The collected bacterial precipitates were immediately liquid nitrogen snap-frozen and stored in the refrigerator at −80°C. The total RNA of these samples was extracted using Trizol reagent (Sigma-Aldrich, United States) according to the specification described method. The RNA concentration and quality were measured using a Nanodrop 2000 micro-spectrophotometer (Thermo Fisher Scientific, Waltham, MA, USA), and agarose gel electrophoresis. RNA-seq library construction and RNA sequencing were performed by the Meiji Biomedical Technology Co., Ltd. (Shanghai, China).

### 2.5 Identification of differentially expressed genes and annotation

The raw data was filtered to remove contaminated and low-quality sequences from connectors and to obtain clean reads. Bowtie2-2.5.0 was used to map the genome of the filtered sequence (Langmead and Salzberg, 2012). The reference genome of *A. baumannii* (ATCC 19606) was downloaded from GenBank (NZ_CP045110.1). The gene expression levels were calculated based on TPM (transcript per million) (Zhao et al., 2021). Differential expression analysis between CRAB77 and CRAB98 was performed using the DEGSeq2 R package. The significance of the DEGs was determined with a *P*-value < 0.05 found by DEseq2. Transcripts with log 2 (Fold Change) > 1 and *P*-value < 0.05 were considered significant differential expressions. KOBAS software was used to test the statistical enrichment of differentially expressed genes in KEGG pathways. Significantly enriched KEGG pathways was identified by a *P*-value < 0.05.

### 2.6 qRT-PCR

qRT-PCR was performed to confirm the DEGs identified using RNA-seq. The primer sequences of candidate genes and a housekeeping gene (16S rRNA) were designed using Primer Premier 5 (Table 4). The first strand of cDNA was synthesized by PrimeScript TMRT reagent Kit with gDNA Eraser (TaKaRa, Beijing, China) according to the manufacturer’s instructions. The qRT-PCR was carried out with a PikoReal 96 real-time PCR system (Thermofisher Scientific) using PowerUp™ SYBR™ Green Master Mix (Thermo Fisher Scientific, Waltham, MA, USA). The sense and anti-sense primers were designed by Primer Premier 5 and they were synthesized by Sangon Biotech Co., Ltd. (Shanghai, China). Sequences of the primers used in this study were shown in detail in Table 4. The reactions were performed with three biological and technical replicates per sample. The data were analyzed using the 2^–ΔCt^ method (Schmittgen and Livak, 2008).

**TABLE 4:** The Primers information for qRT-PCR.

Target gene	Primer name	Primer sequence (5′ → 3′)	Product Size/bp	Tm/°C
*mltG*	mltG-F	CCACCATTACCAGTCGCTACAAAA	180 bp	60
	mltG-R	TGCAAACTATAAGGGGAACATTACTCG		
*dnaK*	dnaK-F	ATTTTTAGGGTTTGTTACTGCCTGA	162 bp	60
	dnaK-R	GGTACTACCAACTCATGTGTTGCTGT		
*groL*	GroL-F	CAGCTGGTTTGTCTTCAGGAATGTC	151	60
	GroL-R	GCAACTGGCGAATATGGTGATATGT		
*FQU82_RS13360*	3360-F	GTTGGATAAGGAACAACACTCACACC	175	60
	3360-R	GTAGCAAAATCTACAACACGCCAAC		
*aceI*	AceI-F	AGTGAAGTGATGCTGCTTTAGCATT	194	60
	AceI-R	TGGTTTTGAGGGTGGTTTGC		
*16S rRNA*	16S rRNA-F	TACACACCGCCCGTCACACC	119	60
	16S rRNA-R	CGGCTACCTTGTTACGACTTCACC		

### 2.7 Statistical analysis

Statistical analysis was performed using SPSS21.0. All experiments were performed in triplicate. Statistical differences between groups were assessed by the two-tailed Student’s *t*-test and one-way analysis of variance (ANOVA) followed by Tukey’s multiple-comparison test or by the nonparametric Mann–Whitney U test and Kruskal-Wallis test, followed by Duncan’s multiple-comparison test. A *P*-value < 0.05 was considered statistically significant.

## 3 Results

### 3.1 Molecular typing of the strains

The sequences of these 7 housekeeping genes in 199 *A. baumannii* strains were submitted to the MLST database of the Pasteur Institute^1^ to investigate whether there were base substitutions or deletions. The sequences obtained from housekeeping gene sequencing of each strain were submitted to the database, and the corresponding alleles of each sequence were queried, to obtain the ST model of each strain. The results showed that there were 9 different sequence types (Table 5), among which ST136, ST191, and ST208 had high frequency in the hospital.

**TABLE 5:** Alleles and sequence types in CRAB isolates.

ST	gltA	gyrB	gdhB	recA	cpn60	gpi	rpoD	Percent (%)
136	1	3	3	2	2	16	3	19.6
191	1	3	3	2	2	94	3	18.6
195	1	3	3	2	2	96	3	15.1
208	1	3	3	2	2	97	3	18.6
2,206	1	63	67	60	29	153	5	0.5
368	1	3	3	2	2	140	3	6
369	1	3	3	2	2	106	3	14.6
457	1	15	3	2	2	153	3	0.5
540	1	3	3	2	2	160	3	6.5

**FIGURE 1:**
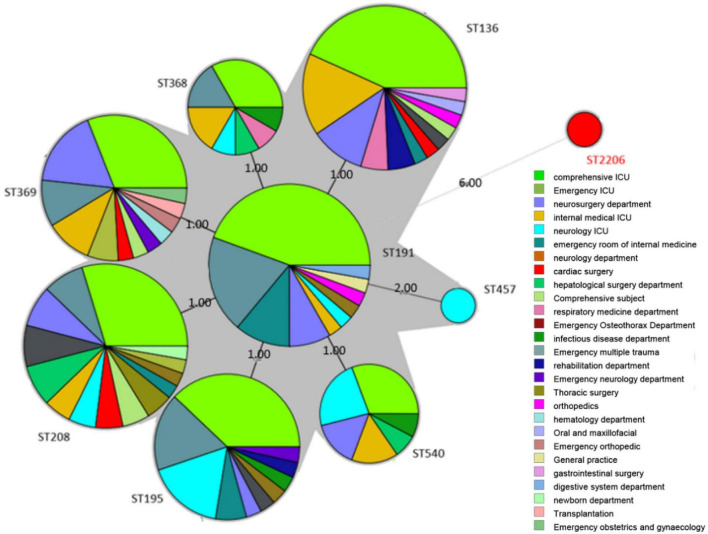
Minimum spanning tree (MST) of 199 *A. baumannii* strains. The size of the circle in MST represents the number of the same ST genotypes, and the black solid line indicates that there are 3 or fewer differences in housekeeping genes between the two ST genotypes. The thicker the black solid line, the smaller the difference in housekeeping genes between the two ST genotypes. The dotted line indicates that there are 4 or more differences in housekeeping genes between the two ST genotypes. The denser the dotted line, the greater the difference in housekeeping genes between ST genotypes.

The minimum spanning tree showed that the ST types of the 199 strains are relatively concentrated, and all of them originated from ST191 (Figure 1). In addition, there is only *gpi* difference between ST136/ST195/ST368/ST369/ST208/ST540 and ST191. While there are two alleles difference between ST457 and ST191, the genetic relationship is close. It is reported that the mutation rate and recombination rate of *gpi* in the MLST typing scheme of *A. baumannii* are higher than those of other housekeeping genes, and it is easy to mutate in the process of evolution. It is found that a similar situation in the study of *A. baumannii* typing, suggests that *A. baumannii* MLST scheme has a better resolution on the housekeeping gene *gpi*. Among the 199 strains of CRAB, there is only one ST, which is widely distributed in various departments. Among these departments, the ICU region is the most distributed. However, the ST2206 does not belong to these ST, and there are six allele differences between ST2206 and ST191, and the ST2206 is isolated in the comprehensive ICU region.

**FIGURE 2:**
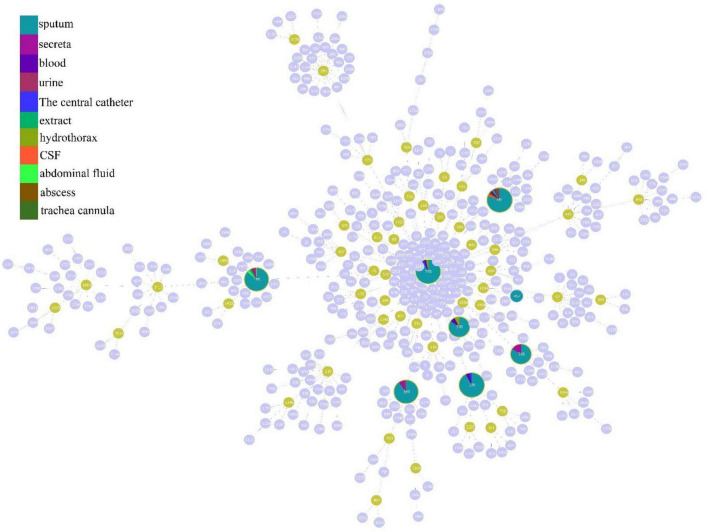
Minimum spanning tree analysis of CRAB isolates based on multilocus sequence typing (MLST) data. Each circle represents an independent sequence type (ST). The size of each circle corresponds to a different number of isolates, with larger sizes representing higher isolate quantity. The lines connecting the circles indicate the relationship between different STs. Different types of lines represent a difference in one allele (solid lines), two alleles (dashed lines), and three or more alleles (dotted lines).

To understand the classification position of these identified strains in the entire *A. baumannii* database, the ST of all strains were placed in the *A. baumannii* ST database. The results showed that the ST136 / ST195 / ST368 / ST369 / ST208 / ST540 / ST191 belongs to CC208, and the ST2206 distant relatives (Figure 2).

**FIGURE 3:**
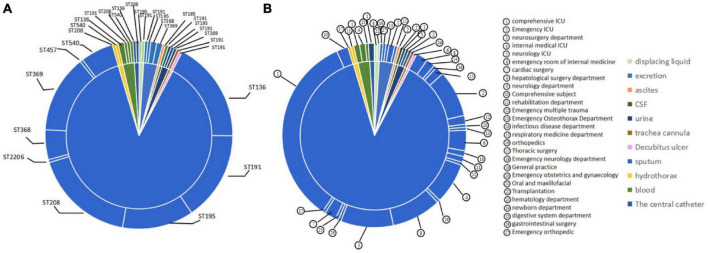
Molecular epidemiological characteristics of strains from different tissue and clinical departments. **(A)** The source of different typing strains. **(B)** The distribution of CRAB strains in sputum in each department.

According to the analysis of the source distribution of 199 CRAB strains, the results showed that most of these clinical strains came from sputum, and a total of 171 strains of each ST type were detected in these sputum samples, while the distribution was less in other samples (Figure 3A). Based on the analysis of sputum samples in various departments, these CRAB were found in all clinical sputum samples in many departments, while there were few types of CRAB samples in the remaining departments (Figure 3B). This result implies that the ST is widely prevalent in all departments in the hospital, and it is necessary to focus on detecting the presence of CRAB in sputum samples to timely control the spread of CRAB.

### 3.2 Antimicrobial susceptibility testing

**FIGURE 4:**
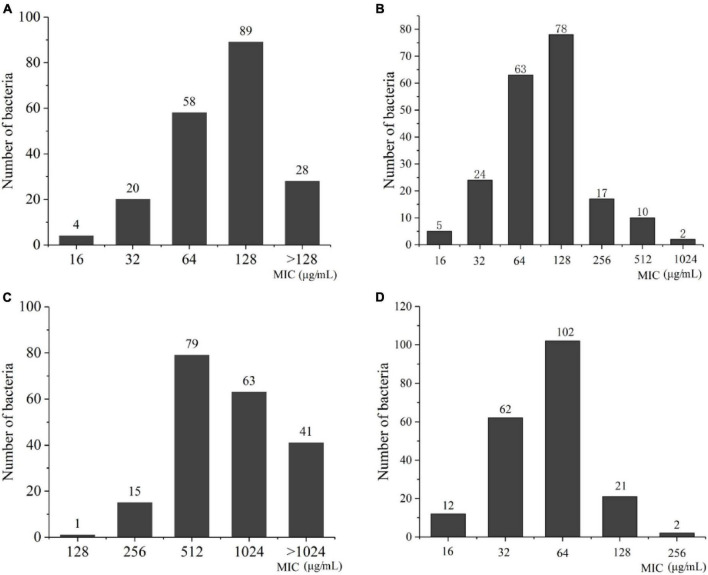
The distribution of MIC value in *A. baumannii*. **(A)** Biapenem; **(B)** meropenem; **(C)** ertapenem; **(D)** imipenem.

The result of MIC values for the 199 *A. baumannii* strains showed that the MIC values for biapenem were concentrated at 128 μg/mL, accounting for 44.5%; the MIC value of meropenem was concentrated at 128 μg/mL, accounting for 39%; the MIC value for ertapenem was concentrated at 512 μg/mL, accounting for 39.5%; as well as the MIC values for imipenem were concentrated at 64 μg/mL, accounting for 51% (Figure 4 and Supplementary Table 1). These results indicate that all experimental strains were highly resistant to the four carbapenems antibiotics, among which the experimental strains had the strongest resistance to ertapenem.

According to the ST type classification of 199 CRAB strains, MIC values of these 199 CRAB strains against ertapenem were calculated. The results showed that ST136, ST191, ST195, and ST208 were more resistant to ertapenem than ST368, ST369, ST457, and ST540. However, the ST2206, which is distantly related to STs strain, also had a MIC value of 1,024 μg/mL. This result indicates that ST2206 with strong antibiotic resistance (Table 6).

**TABLE 6:** Resistance of ST types to ertapenem in 199 CRAB strains.

	Ertapenem MIC (μg/mL)					
ST	<32	128	256	512	1,024	>1,024
ST136	0	0	2	9	14	14
ST191	0	0	0	14	13	10
ST195	0	0	3	9	13	5
ST208	0	1	1	19	10	6
ST2206	0	0	0	0	1	0
ST368	0	0	4	4	4	0
ST369	0	0	4	15	4	6
ST457	0	0	0	0	1	0
ST540	0	0	1	9	3	0

In addition, according to the antibiotic resistance detection of these 199 strains to biapenem, the results showed that the MIC values of ST types concentrated at 128 μg/mL. The result showed that these ST types have the same resistance to biapenem. However, the ST2206 showed lower resistance to biapenem than other ST types (Table 7).

**TABLE 7:** Resistance of ST types to biapenem in 199 CRAB strains.

	Biapenem MIC (μg/mL)				
ST	16	32	64	128	>128
ST136	1	3	8	17	10
ST191	0	1	10	24	2
ST195	0	3	9	12	6
ST208	2	2	15	14	4
ST2206	0	1	0	0	0
ST368	1	3	2	5	1
ST369	0	4	9	11	5
ST457	0	0	0	1	0
ST540	0	3	5	5	0

According to the detection of the resistance for these 199 CRAB strains to meropenem, it was found that the MIC values of STs to meropenem in these 199 CRAB strains were higher, while the ST2206 showed low resistance to meropenem (Table 8).

**TABLE 8:** Resistance of ST types to meropenem in 199 CRAB strains.

	Meropenem MIC (μg/mL)						
ST	16	32	64	128	256	512	>512
ST136	0	5	4	24	3	2	1
ST191	0	2	6	21	8	0	0
ST195	0	4	8	13	3	1	1
ST208	1	7	18	9	1	1	0
ST2206	0	0	1	0	0	0	0
ST368	3	3	2	4	0	0	0
ST369	0	3	16	5	2	3	0
ST457	0	0	0	1	0	0	0
ST540	1	0	8	1	0	3	0

Based on the detection of the resistance STs to imipenem in 199 CRAB strains, the results showed that MIC values were concentrated in 32 μg/mL-128 μg/mL, indicating strong resistance. Moreover, the ST2206 also showed resistance to imipenem, but the resistance was low, with a MIC value of 32 μg/mL (Table 9). According to this data, it was be defined weakly resistant strain with MIC values ≤ 32 to imipenem, while strains with MIC value > 32 was defined as strongly resistant strain. By this definition, ST136 and ST208, have quite different resistance to imipenem; In this study, weakly resistant strains accounted for 18% in ST136, and 47.22% were weakly resistant strains in ST208. This result indicates that different STs may have different resistance mechanisms to imipenem.

**TABLE 9:** Resistance of ST types to imipenem in 199 CRAB strains.

	Imipenem MIC (μg/mL)					
ST	<16	16	32	64	128	256
ST136	0	0	7	25	6	1
ST191	0	0	4	21	11	1
ST195	1	1	11	17	0	0
ST208	5	0	14	17	1	0
ST2206	0	0	1	0	0	0
ST368	3	0	4	5	0	0
ST369	0	0	12	14	3	0
ST457	0	0	0	1	0	0
ST540	2	0	9	2	0	0

### 3.3 Antimicrobial resistance genes testing

**FIGURE 5:**
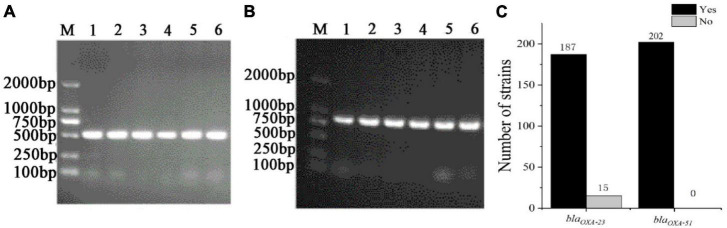
The PCR amplification of CRAB (part of the sample) resistance genes and the statistics of positive tests of antibiotic resistance genes. **(A)** *bla*_*OXA*–23_; **(B)** *bla*_*OXA*–51_; **(C)** Statistics of positive tests of antibiotic resistance genes; M, DNA maker; Yes, the number of positive strains; no, the number of negative strains.

To understand the distribution of antibiotic resistance genes, the *bla*_*OXA*–23_, *and bla_*OXA–51*_* were detected in 199 CRAB, standard *A. baumannii* (ATCC19606), pan-resistant *A. baumannii* (PRAB) and SJZ24 by using PCR amplification. The results showed that the target sequence of *bla*_*OXA*–23_ (Figure 5A) and *bla*_*OXA*–51_ (Figure 5B) genes were found in these strains. The BLAST analysis was conducted after sequencing these target bands, and it was found that these target bands were all target antibiotic resistance genes. Statistical data showed that the detection rate of the *bla*_*OXA*–23_ gene and *bla*_*OXA*–51_ gene in CRAB was 92.5 and 100%, respectively (Figure 5C). These results suggest that the class D carbapenemases genes are widely distributed in these 199 CRAB strains, which may be one of the reasons for its high antibiotic resistance to carbapenems antibiotics.

### 3.4 Transcriptome profiling and identifying differentially expressed genes (DEGs)

**FIGURE 6:**
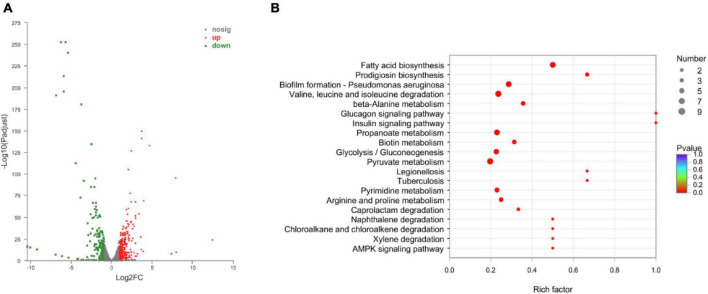
The Volcano diagram of differentially expressed genes between resistance strain and sensitive strain **(A)** and DEGs involved in KEGG pathway enrichment analysis in comparison with resistance strain and sensitive strain **(B)**. In **(A)**, The *x*-axis represents the multiple differential expressions; the *y*-axis represents significance. In **(B)**, The *x*-axis represents the Rich factor, GeneRatio = Term Candidate Gene Number/Term Gene Number. The *y*-axis represents KEGG Pathway. The size of the bubble is proportional to the number of genes in the KEGG Pathway. And the color represents the *P*-value of enrichment. The deeper the color, the smaller the *P*-value.

To understand the resistance mechanisms of different STs to imipenem, the transcriptome sequencing analysis between ST208 (AB77) and ST136 (AB98) was performed in this study. The results showed that a sum of 159.60 million reads was generated and each sample provided an average production of 26.60 million. A mean of 26.60 million clean reads was obtained from each library with an adequate read ratio of 94.63% after removing adaptor sequences, N-containing reads, and low-quality. A mean of 20.44 million CDS-mapped reads was obtained from each library with an adequate read ratio of 80.11% (Supplementary Table 2). A total of 3,829 genes were mapped from all the samples. The resistance strain and sensitive strain gene expression levels were analyzed, and 390 differentially expressed genes (DEGs) were recognized (Supplementary Table 3). Among such DEGs, the resistance strain had 256 DEGs up-regulated and 134 DEGs down-regulated (Figure 6A). It is worth pointing out that some genes related to antibiotic resistance, efflux pump, and biofilm formation were up-regulated in the resistance strain (Figure 6B). The increased expression level of these genes may increase the antibiotic resistance of *A. baumannii*.

### 3.5 Resistance genes regulated antibiotic resistance

Among these DEGs, 19 genes may be involved the carbapenem resistance, including biofilm formation, efflux pump, peptidoglycan biosynthesis, chaperonin synthesis, and drug transporter. In the biofilm formation process, there were pilus assembly protein (*Pap*, *FQU82_RS03605*), urease accessory protein (*UreD*, *FQU82_RS05390*), OmpA family protein (*FQU82_RS07900*), NADP-specific glutamate dehydrogenase (*gdhA*), phosphoenolpyruvate–protein phosphotransferase (*pstP*), and amido phosphoribosyl transferase (*purF*, *FQU82_RS13115*). In addition, there were 5 genes involved in efflux pump, including LysR family transcriptional regulator (*FQU82_RS08650*), PACE efflux transporter (*FQU82_RS09170*), chlorhexidine efflux PACE transporter (*aceI*, *FQU82_RS12055*), MacB family efflux pump subunit (*FQU82_RS03105*), MacA family efflux pump subunit (*FQU82_RS03110*). Meanwhile, there were 3 genes may contribute to peptidoglycan biosynthesis, including D-alanyl-D-alanine carboxypeptidase PBP5/6 (*dacC*, *FQU82_RS14285*), endocytic transglycosylase (MltG, *FQU82_RS14770*), and UDP-glucose 4-epimerase (*GalE*, *FQU82_RS00890*). Additionally, there were 3 chaperonins, including *DnaK*, *GroEL*, and *GroES* may be involved in antibiotic resistance. It is worth mentioning that the MFS transporter and ABC transporter substrate-binding protein may participate in drug transport (Table 10).

**TABLE 10:** *A. baumannii* genes involved in antibiotic resistance identified by mRNA-seq.

Gene id	Gene name	Gene function	Log2FC	q value
FQU82_RS03605	*FQU82_RS03605*	Pilus assembly protein	1.121204	0.0441
FQU82_RS05390	*FQU82_RS05390*	Urease accessory protein UreD	1.87116	0.0000
FQU82_RS05470	*gdhA*	NADP-specific glutamate dehydrogenase	1.067204	0.0000
FQU82_RS07900	*FQU82_RS07900*	OmpA family protein	-2.33007	0.0000
FQU82_RS02520	*ptsP*	Phosphoenolpyruvate–protein phosphotransferase	-1.01688	0.0000
FQU82_RS13115	*purF*	amidophosphoribosyltransferase	1.175621	0.0000
FQU82_RS14285	*dacC*	D-alanyl-D-alanine carboxypeptidase PBP5/6	1.375552	0.0000
FQU82_RS14770	*mltG*	Endocytic transglycosylase MltG	1.084947	0.0000
FQU82_RS00890	*galE*	UDP-glucose 4-epimerase GalE	-1.13269	0.0000
FQU82_RS00325	*dnaK*	Molecular chaperone DnaK	1.044462	0.0000
FQU82_RS15275	*groL*	Chaperonin GroEL	1.287527	0.0000
FQU82_RS15280	*FQU82_RS15280*	Co-chaperone GroES	3.371287	0.0000
FQU82_RS03755	*FQU82_RS03755*	MFS transporter	1.581374	0.0000
FQU82_RS13360	*FQU82_RS13360*	ABC transporter substrate-binding protein	1.286576	0.0000
FQU82_RS08650	*FQU82_RS08650*	LysR family transcriptional regulator	-1.09716	0.0000
FQU82_RS09170	*FQU82_RS09170*	PACE efflux transporter	1.446246	0.0000
FQU82_RS12055	*aceI*	Chlorhexidine efflux PACE transporter AceI	1.787986	0.0000
FQU82_RS03105	*FQU82_RS03105*	MacB family efflux pump subunit	-2.07775	0.0000
FQU82_RS03110	*FQU82_RS03110*	MacA family efflux pump subunit	-2.5575	0.0000

### 3.6 Verification of differential expression gene by quantitative real-time PCR

**FIGURE 7:**
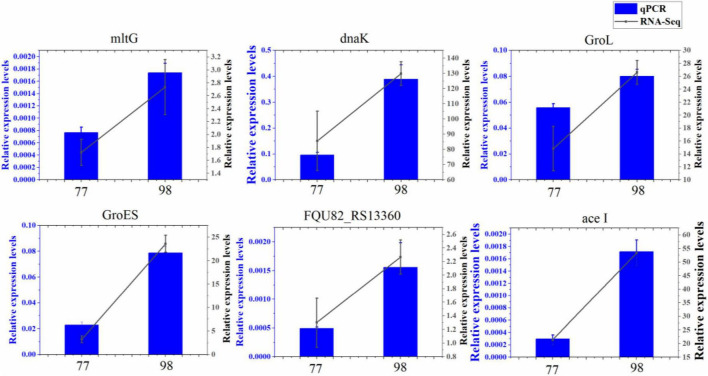
Relative expression levels of 6 DEGs in 77 and 98. *MltG*, endolytic transglycosylase MltG; *dnaK*, molecular chaperone DnaK; *GroL*, chaperonin GroEL; *GroES*, co-chaperone GroES; *FQU82_RS13360*, ABC transporter substrate-binding protein; *ace I*, chlorhexidine efflux PACE transporter AceI.

To confirm findings of gene expression obtained from transcriptome data, 6 DEGs concerned with antibiotic resistance were chosen for qRT-PCR. These DEGs are mainly involved in biofilm formation, efflux pump, peptidoglycan biosynthesis, and chaperonin synthesis. As shown in, the 6 DEGs had a very similar expression pattern based on the transcriptome data and qRT-PCR, which indicates the trustworthiness of the transcriptomic analysis (Figure 7).

## 4 Discussion

*A. baumannii* is mainly related to nosocomial infections globally and is an oxybiotic Gram-negative pathogen that results in infections of the skin, urinary tract, bloodstream, and other soft tissues (Lee et al., 2017). In recent years, *A. baumannii* have become a significant cause of critical healthcare-associated diseases and infections (Lima et al., 2019). Among these β-lactam antibiotics, the carbapenems are considered the most effective class with the most extensive spectrum of antimicrobial activity (O’Donnell et al., 2021). Hence, carbapenem antibiotics are also often used in the treatment of *A. baumannii* infection (Nguyen and Joshi, 2021). However, with the use of carbapenem antibiotics, some carbapenem-resistant bacteria, including *A. baumannii*, *Pseudomonas aeruginosa*, and *Stenotrophomonas maltophilia* were appeared (Nordmann and Poirel, 2019). It is reported that *A. baumannii* infections occur in critically ill patients in the ICU setting and account for up to 20% of infections in ICUs worldwide (Lee et al., 2017). To better understand the carbapenem resistance *A. baumannii* to be able to develop a good treatment plan. In this study, 199 CRAB strains were used as materials to study their typing. Among the 199 CRAB strains, two strains were used to explore the mechanism of antibiotic resistance.

According to the MLST analysis of the 199 CRAB strains, it was found that the ST136 and ST208 were the most STs of CRAB, which have strong resistance to the four kinds of carbapenem antibiotics. As in previous studies, the ST136 and ST208 belong to CC92, which is the most widely and largest disseminated complex worldwide (Alcantar-Curiel et al., 2019; Lima et al., 2019). Additionally, ST208 has become the main sequence type of *A. baumannii* infection outbreak in western China (Qu et al., 2016; Gao et al., 2022). Furthermore, along with the global phenomenon, CC92 has become the dominant circulating strain in children (Kang et al., 2023). In addition, the present study showed that the ST195, ST191, and ST369 were divided into the same group, while the time of emergence of these two STs was different. It is worth noting that the virulence of ST369 is higher than that of ST191 (Jiang et al., 2021). Moreover, the resistance of ST191 to carbapenem is also very serious (Tables 6–9). Therefore, it is extremely important to control the spread of ST136, ST191, ST208, ST195, ST369.

Imipenem is a carbapenem antibiotic commonly used in clinical treatment and plays an important role in preventing *A. baumannii* infection (Khaled et al., 2021). A large number of studies have shown that *A. baumannii* ST136 is highly resistant to imipenem (Firoozeh et al., 2023; You et al., 2023). In the present study, ST136 also showed strong resistance to imipenem, while ST208 showed weak resistance to imipenem (Table 9). This phenomenon may be caused by differences in gene expression at different STs. Transcriptome results showed that these two STs *A. baumannii* were significantly differentially expressed in biofilm formation, efflux pump, peptidoglycan biosynthesis, and chaperone biosynthesis. These results suggest that the resistance ability of ST136 to imipenem is caused by the differential gene expression of these pathways.

As we all know, bacterial resistance to antibiotics involves many factors, such as the formation of biofilm, which can increase antibiotic resistance thousands of times (Guo et al., 2022). Additionally, the efflux pump, the chaperonins, and peptidoglycan biosynthesis also play important roles in antibiotic resistance (Grishin et al., 2020; Stevens et al., 2020; Barnabas et al., 2022). During the biofilm formation, the pilus assembly protein plays an important role in the formation of fimbriae (Sanchez et al., 2017; Shanmugasundarasamy et al., 2022). In this study, the pilus assembly protein was up-regulated in the antibiotic resistance strains (Table 6). In addition, the *ureD* (Maisuria et al., 2015), *ptsP* (Abisado et al., 2021), and *purF* (Li et al., 2021) also contribute to biofilm formation, which may play an important in the process of antibiotic resistance. In the meanwhile, a *ureD*, and *ptsP* were also up-regulated in this study (Table 10). These results suggest that biofilm formation plays an important role in carbapenem antibiotic resistance.

To date, different categories of efflux pumps have been identified in *A. baumannii*, including RND-family (resistance-nodulation-division), MFS (major facilitator superfamily), MATE (multidrug and toxic compound extrusion), SMR (small multi antibiotic resistance), PACE family and ABC (ATP-binding cassette) superfamilies are the main components of the efflux pump (Blair et al., 2014; Li et al., 2015; Hassan et al., 2018). These efflux pumps play a key role in antibiotic resistance (Roy et al., 2022). In this study, the MFS transporter, and PACE efflux transporter were up-regulated (Table 10), which may improve the ability of carbapenem antibiotic resistance. Most importantly, LysR, a transcriptional regulator, also indirectly regulates the efflux pump (Fu et al., 2020; Lu et al., 2021), which is also significantly up-regulated in this study.

Besides, the peptidoglycan also contributes to antibiotic resistance (Kvesić et al., 2022). It is reported that the deposition of *Pseudomonas aeruginosa* with the interference of peptidoglycan circulation in the lungs of mice was significantly reduced, and the mortality caused by systemic infection was significantly reduced (Torrens et al., 2019). Moreover, the peptidoglycan synthesis related gene *dacC* was more frequently found in populations with lower relative fitness across the measured growth parameters (Barbosa et al., 2017). In the meanwhile, the *dacC* in the antibiotic resistance strain was upregulated (Table 10), indicating that *dacC* may also be involved in antibiotic resistance.

**FIGURE 8:**
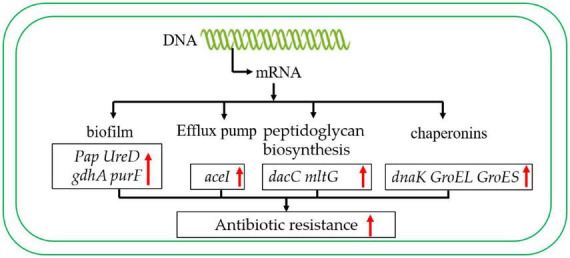
Carbapenem-resistant *A. baumannii* antibiotic resistance regulation model. The red arrow indicates that the expression level was up-regulated.

As in previous studies, chaperone proteins also play an important role in antibiotic resistance (Chiang et al., 2022). For instance, in *Escherichia coli*, the Chaperonin *GroEL/GroES* over-expression can promote aminoglycoside resistance and reduce drug susceptibilities (Goltermann et al., 2015). Moreover, the *GroEL* can increase the antibiotic resistance of *Klebsiella pneumoniae* by promoting the refolding of misfolded proteins (Ye et al., 2021). It is worth mentioning that the chaperone GroEL/GroES of *A. baumannii* has also been reported to play an important role in antimicrobial resistance (Abirami et al., 2023). In addition, the DnaK system supports rifampicin resistance (Fay et al., 2021). In this study, the chaperone proteins *GroEL*, *GroES* and *DnaK* are up-regulated in the antibiotic-resistance strain. These results suggest that chaperones may also play a crucial role in the process of carbapenem antibiotic resistance (Figure 8).

In summary, these results showed that the main STs of CRAB were ST136, ST191, ST208, ST195, ST369, and it was mainly distributed in ICU. The analysis of the antibiotic resistance mechanism suggested that the antibiotic resistance mechanism of *A. baumannii* was mainly due to the expression of biofilm formation and efflux pump-related genes. In addition, to peptidoglycan biosynthesis, chaperonin synthesis also plays an important role. This study provides a theoretical basis for better control of CRAB.

## 5 Conclusion

In this study, there were 9 ST sequence types were detected, and the CC208 was the main clonal complex in this hospital and showed high carbapenems resistance; as well as the *bla*_*OXA*–23_ gene was the main carbapenems resistance genotypes; the results of transcriptome data showed that the mechanism of enhanced carbapenems resistance of *A. baumannii* may be due to the enhanced biofilm-forming ability, efflux pump expression, peptidoglycan biosynthesis, and molecular chaperone protein expression. Therefore, we need to strengthen the research and development of new antibiotics to provide an effective way to control carbapenem-resistant *A. baumannii*. This study lays the foundation for the detection and control of carbapenem *A. baumannii*. In summary, the epidemic trend of clinical *A. baumannii* in Guiyang, China was analyzed from the molecular level, and the antibiotic resistance mechanism of *A. baumannii* to carbapenem antibiotics was analyzed with transcriptome, which provided a theoretical basis for better control of *A. baumannii*.

## Data availability statement

The original contributions presented in this study are included in this article/Supplementary material, further inquiries can be directed to the corresponding authors.

## Author contributions

ZQ: Funding acquisition, Methodology, Resources, Writing – original draft. KY: Methodology, Software, Writing – original draft. HC: Methodology, Software, Writing – original draft. SC: Methodology, Software, Writing – review & editing. FC: Writing – review & editing. FM: Funding acquisition, Writing – review & editing: GG: Funding acquisition, Writing – review & editing. JP: Funding acquisition, Resources, Writing – review & editing.

## Footnotes

1
https://pubmlst.org/abaumannii/
