# Supplementary figures and images for: Cross-talk of MLST and transcriptome unveiling antibiotic resistance mechanism of carbapenem resistance Acinetobacter baumannii clinical strains isolated in Guiyang, China
Source: Front Microbiol. 2024 Jun 14;15:1394775. doi: 10.3389/fmicb.2024.1394775 (PMC11211267; doi:10.3389/fmicb.2024.1394775)

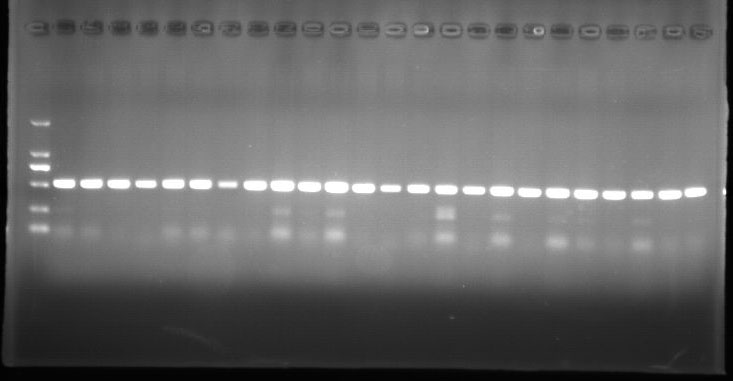

Supplement: Supplementary file 2 [file Data_Sheet_2.ZIP › Source data/Antibiotic resistance gene gel/OXA23.jpg]

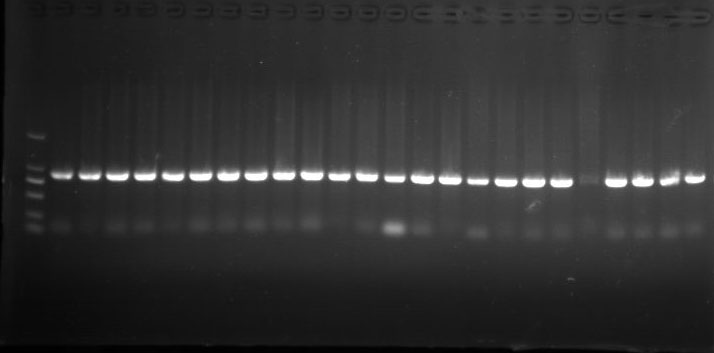

Supplement: Supplementary file 2 [file Data_Sheet_2.ZIP › Source data/Antibiotic resistance gene gel/OXA51.jpg]
